# *catena*-Poly[[[aqua­(5-nitro­benzene-1,2,3-tricarboxyl­ato-κ*O* ^1^)nickel(II)]-di-μ-aqua-[diaqua­sodium]-di-μ-aqua] tetra­hydrate]

**DOI:** 10.1107/S1600536810006872

**Published:** 2010-02-27

**Authors:** Zheng-De Tan, Bing Yi

**Affiliations:** a College of Chemistry and Chemical Engineering, Hunan Institute of Engineering, Xiang Tan 411104, People’s Republic of China

## Abstract

In the title complex, {[NaNi(C_9_H_2_NO_8_)(H_2_O)_7_]·4H_2_O}_*n*_, the Ni^II^ atom has a distorted octa­hedral coordination, defined by five O atoms from five water mol­ecules and one O atom from one 5-nitro­benzene-1,2,3-tricarboxyl­ate ligand. The Na cation is coordinated by six water O atoms in an irregular trigonal-prismatic geometry. There are seven coordinated water mol­ecules in the asymmetic unit. The Ni and Na atoms are linked by water bridges, forming infinite chains, which are connected by strong O—H⋯O hydrogen bonds involving the coordinated and uncoordinated water mol­ecules into a three-dimensional network.

## Related literature

For related structures, see: Ding & Zhao (2010 ▶); Li *et al.* (2006 ▶).
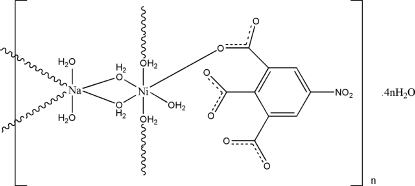


## Experimental

### 

#### Crystal data

[NaNi(C_9_H_2_NO_8_)(H_2_O)_7_]·4H_2_O
*M* *_r_* = 531.99
Triclinic, 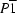

*a* = 6.7005 (6) Å
*b* = 13.161 (4) Å
*c* = 13.586 (4) Å
α = 63.415 (6)°
β = 79.076 (6)°
γ = 81.857 (6)°
*V* = 1049.8 (4) Å^3^
*Z* = 2
Mo *K*α radiation
μ = 1.04 mm^−1^
*T* = 296 K
0.32 × 0.30 × 0.21 mm

#### Data collection

Bruker APEXII area-detector diffractometer
Absorption correction: multi-scan (*SADABS*; Sheldrick, 2005 ▶) *T*_min_ = 0.733, *T*_max_ = 0.812
5287 measured reflections
3702 independent reflections
3115 reflections with *I* > 2σ(*I*)
*R*_int_ = 0.025

#### Refinement

*R*[*F* ^2^ > 2σ(*F* ^2^)] = 0.052
*wR*(*F* ^2^) = 0.172
*S* = 0.82
3702 reflections
280 parameters
33 restraints
H-atom parameters constrained
Δρ_max_ = 0.85 e Å^−3^
Δρ_min_ = −1.02 e Å^−3^

### 

Data collection: *APEX2* (Bruker, 2004 ▶); cell refinement: *SAINT* (Bruker, 2004 ▶); data reduction: *SAINT*; program(s) used to solve structure: *SHELXS97* (Sheldrick, 2008 ▶); program(s) used to refine structure: *SHELXL97* (Sheldrick, 2008 ▶); molecular graphics: *XP* in *SHELXTL* (Sheldrick, 2008 ▶); software used to prepare material for publication: *SHELXTL*.

## Supplementary Material

Crystal structure: contains datablocks I, global. DOI: 10.1107/S1600536810006872/si2240sup1.cif

Structure factors: contains datablocks I. DOI: 10.1107/S1600536810006872/si2240Isup2.hkl

Additional supplementary materials:  crystallographic information; 3D view; checkCIF report

## Figures and Tables

**Table 1:** Hydrogen-bond geometry (Å, °)

*D*—H⋯*A*	*D*—H	H⋯*A*	*D*⋯*A*	*D*—H⋯*A*
O8*W*—H16*W*⋯O6*W*^i^	0.84	2.07	2.876 (4)	161
O8*W*—H15*W*⋯O2^ii^	0.84	2.06	2.835 (4)	153
O7*W*—H13*W*⋯O10*W*^iii^	0.84	1.93	2.748 (4)	163
O7*W*—H14*W*⋯O1^ii^	0.84	1.90	2.731 (4)	172
O10*W*—H20*W*⋯O6	0.84	1.92	2.735 (5)	165
O10*W*—H19*W*⋯O9*W*	0.84	2.01	2.813 (4)	160
O9*W*—H18*W*⋯O3^iv^	0.84	1.99	2.802 (4)	161
O9*W*—H17*W*⋯O4	0.84	1.89	2.734 (4)	176
O11*W*—H22*W*⋯O7*W*	0.84	1.85	2.676 (4)	168
O11*W*—H21*W*⋯O10*W*^v^	0.84	1.95	2.784 (4)	173
O1*W*—H2*W*⋯O4^vi^	0.84	1.89	2.721 (3)	172
O1*W*—H1*W*⋯O9*W*^v^	0.84	1.86	2.685 (4)	168
O3*W*—H6*W*⋯O8*W*	0.84	1.85	2.660 (4)	162
O3*W*—H5*W*⋯O2^vi^	0.84	1.97	2.783 (4)	162
O2*W*—H4*W*⋯O3^vi^	0.84	1.83	2.657 (3)	170
O2*W*—H3*W*⋯O4	0.84	2.00	2.826 (4)	168
O4*W*—H7*W*⋯O6	0.84	1.84	2.655 (4)	163
O4*W*—H8*W*⋯O7*W*	0.84	2.02	2.801 (4)	154
O6*W*—H11*W*⋯O8*W*	0.84	2.09	2.900 (5)	161
O6*W*—H12*W*⋯O1^vii^	0.84	2.11	2.919 (4)	160
O5*W*—H9*W*⋯O3^vi^	0.84	2.25	2.935 (4)	139
O5*W*—H10*W*⋯O2^viii^	0.84	2.18	2.913 (5)	145

Symmetry codes: (i) 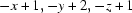; (ii) 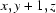; (iii) 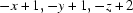; (iv) 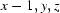; (v) 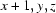; (vi) 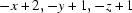; (vii) 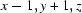; (viii) 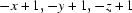.

**Fig. 1.**
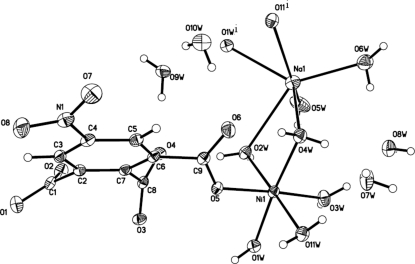
A section of the structure of the title compound, showing the atomic numbering scheme with 30% probability displacement ellipsoids. [Symmetry codes: (i) x-1, y, z.]

**Fig. 2.**
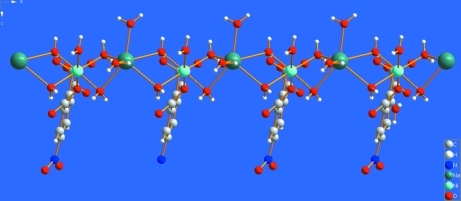
The chain of the title compound along the b axis (the uncoordinated water molecules have been omitted for clarity).

## supplementary crystallographic information

### Comment

It is well-known that carboxylate ligands play an important role in coordination chemistry. They usually adopt diverse binding modes as terminal monodentate, chelating to one metal center, bridging to two metal centers (Ding *et al.*, 2010; Li *et al.*, 2006). In the present paper, we synthesized a novel green complex {[NiNa(C_9_H_2_NO_8_)(H_2_O)_7_].4(H_2_O)}_n_ based on 5-nitrobenzene-1,2,3-tricarboxylate ligand. It is isostructural to the copper compound reported by Ding & Zhao, (2010).

The coordination geometries of Ni and Na centers are very close to the values observed in the {[CuNa(C_9_H_2_NO_8_)(H_2_O)_7_].4(H_2_O)}_n_ compound, and also the hydrogen bonds in both structures are very similar (Fig.1). The Ni and Na atoms are linked by water bridges, forming an infinite chain (Fig.2). They are arrayed by turns with the distance of 3.4258 (21)Å and 3.7373 (20)Å between Ni and Na. The chains are connected by strong O—H···O hydrogen bonds involving the coordinated and uncoordinated water molecules into a three-dimensional network (Table 1). In the {[CuNa(C_9_H_2_NO_8_)(H_2_O)_7_].4(H_2_O)}_n_ compound, the six Cu—O bond lengths range between 2.028 (2) and 2.098 (3) Å. It is a rare case that all Cu—O distances are above 2.00 Å, which may be explained by the influence of the Na—O—Cu bridges. But in the title compound, the Ni—O bond lengths range between 2.032 (2) and 2.106 (3) Å and represent normal values.

### Experimental

A mixture of 5-nitrobenzene-1,2,3-tricarboxylate ligand (0.1 mmol), Ni(NO_3_)_2_ (0.1 mmol) and H_2_O (20 ml) was treated with a solution of NaOH until the pH about 7-8. and left to stand at room temperature for about a few weeks,then the green crystals were obtained.

### Refinement

Carbon bound H atoms were placed at calculated positions and were treated as riding on the parent C atoms with C—H = 0.93 Å, and with *U*_iso_(H) = 1.2 *U*_eq_(C). The water H-atoms were located in a difference map, and were refined with a distance restraint of O—H = 0.84 Å; their *U*_iso_ values were refined.

### Crystal data

**Table:**

[NaNi(C_9_H_2_NO_8_)(H_2_O)_7_]·4H_2_O	*Z* = 2
*M**_r_* = 531.99	*F*(000) = 552
Triclinic, *P*1	*D*_x_ = 1.683 Mg m^−^^3^
Hall symbol: -P 1	Mo *K*α radiation, λ = 0.71073 Å
*a* = 6.7005 (6) Å	Cell parameters from 2858 reflections
*b* = 13.161 (4) Å	θ = 3.1–28.2°
*c* = 13.586 (4) Å	µ = 1.04 mm^−^^1^
α = 63.415 (6)°	*T* = 296 K
β = 79.076 (6)°	Block, green
γ = 81.857 (6)°	0.32 × 0.30 × 0.21 mm
*V* = 1049.8 (4) Å^3^	

### Data collection

**Table:**

Bruker APEXII area-detector diffractometer	3702 independent reflections
Radiation source: fine-focus sealed tube	3115 reflections with *I* > 2σ(*I*)
graphite	*R*_int_ = 0.025
φ and ω scan	θ_max_ = 25.2°, θ_min_ = 1.7°
Absorption correction: multi-scan (*SADABS*; Sheldrick, 2005)	*h* = −8→7
*T*_min_ = 0.733, *T*_max_ = 0.812	*k* = −11→15
5287 measured reflections	*l* = −16→15

### Refinement

**Table:**

Refinement on *F*^2^	Primary atom site location: structure-invariant direct methods
Least-squares matrix: full	Secondary atom site location: difference Fourier map
*R*[*F*^2^ > 2σ(*F*^2^)] = 0.052	Hydrogen site location: inferred from neighbouring sites
*wR*(*F*^2^) = 0.172	H-atom parameters constrained
*S* = 0.82	*w* = 1/[σ^2^(*F*_o_^2^) + (0.162*P*)^2^ + 0.8519*P*] where *P* = (*F*_o_^2^ + 2*F*_c_^2^)/3
3702 reflections	(Δ/σ)_max_ = 0.001
280 parameters	Δρ_max_ = 0.85 e Å^−^^3^
33 restraints	Δρ_min_ = −1.02 e Å^−^^3^

### Special details

**Table:**

Geometry. All esds (except the esd in the dihedral angle between two l.s. planes) are estimated using the full covariance matrix. The cell esds are taken into account individually in the estimation of esds in distances, angles and torsion angles; correlations between esds in cell parameters are only used when they are defined by crystal symmetry. An approximate (isotropic) treatment of cell esds is used for estimating esds involving l.s. planes.
Refinement. Refinement of *F*^2^ against ALL reflections. The weighted *R*-factor wR and goodness of fit *S* are based on *F*^2^, conventional *R*-factors *R* are based on *F*, with *F* set to zero for negative *F*^2^. The threshold expression of *F*^2^ > σ(*F*^2^) is used only for calculating *R*-factors(gt) etc. and is not relevant to the choice of reflections for refinement. *R*-factors based on *F*^2^ are statistically about twice as large as those based on *F*, and *R*- factors based on ALL data will be even larger.

### Fractional atomic coordinates and isotropic or equivalent isotropic displacement parameters (Å^2^)

**Table:**

	*x*	*y*	*z*	*U*_iso_*/*U*_eq_	
C1	0.9163 (6)	0.0491 (3)	0.7521 (3)	0.0269 (8)	
C2	0.8515 (5)	0.1016 (3)	0.8336 (3)	0.0228 (7)	
C3	0.7844 (6)	0.0308 (3)	0.9447 (3)	0.0252 (8)	
H3	0.7885	−0.0476	0.9691	0.030*	
C4	0.7123 (5)	0.0780 (3)	1.0174 (3)	0.0248 (8)	
C5	0.7116 (5)	0.1939 (3)	0.9862 (3)	0.0252 (8)	
H5	0.6619	0.2237	1.0372	0.030*	
C6	0.7864 (5)	0.2642 (3)	0.8773 (3)	0.0232 (8)	
C7	0.8516 (5)	0.2191 (3)	0.7993 (3)	0.0226 (8)	
C8	0.9163 (6)	0.2966 (3)	0.6771 (3)	0.0232 (7)	
C9	0.7955 (6)	0.3896 (3)	0.8445 (3)	0.0247 (8)	
N1	0.6278 (5)	0.0040 (3)	1.1322 (2)	0.0304 (7)	
Na1	0.4252 (3)	0.71871 (14)	0.62371 (12)	0.0367 (4)	
Ni1	0.95998 (7)	0.61127 (4)	0.67564 (3)	0.0235 (2)	
O1	1.0145 (5)	−0.0455 (2)	0.7877 (2)	0.0384 (7)	
O2	0.8649 (4)	0.1028 (2)	0.6566 (2)	0.0344 (7)	
O3	1.0990 (4)	0.2896 (2)	0.6371 (2)	0.0292 (6)	
O4	0.7775 (4)	0.3631 (2)	0.62481 (19)	0.0278 (6)	
O5	0.9363 (4)	0.4410 (2)	0.7681 (2)	0.0252 (6)	
O6	0.6652 (5)	0.4346 (2)	0.8957 (2)	0.0395 (7)	
O7	0.5232 (5)	0.0489 (3)	1.1881 (2)	0.0468 (8)	
O8	0.6619 (5)	−0.0989 (2)	1.1654 (2)	0.0440 (8)	
O5W	0.5203 (6)	0.8170 (3)	0.4318 (3)	0.0609 (10)	
H10W	0.4216	0.8129	0.4041	0.091*	
H9W	0.6299	0.8222	0.3885	0.091*	
O6W	0.3273 (5)	0.8978 (3)	0.6356 (3)	0.0474 (8)	
H12W	0.2531	0.9289	0.6730	0.071*	
H11W	0.4516	0.9004	0.6361	0.071*	
O4W	0.6914 (4)	0.6549 (2)	0.7614 (2)	0.0344 (6)	
H8W	0.7288	0.6999	0.7818	0.052*	
H7W	0.6762	0.5899	0.8144	0.052*	
O2W	0.7733 (4)	0.5951 (2)	0.5785 (2)	0.0286 (6)	
H3W	0.7918	0.5254	0.5944	0.043*	
H4W	0.7993	0.6346	0.5096	0.043*	
O3W	1.0007 (5)	0.7794 (2)	0.5696 (2)	0.0362 (7)	
H5W	1.0372	0.8014	0.5009	0.054*	
H6W	0.9399	0.8326	0.5838	0.054*	
O1W	1.2328 (4)	0.5797 (2)	0.5931 (2)	0.0294 (6)	
H1W	1.2943	0.5159	0.6247	0.044*	
H2W	1.2408	0.5999	0.5245	0.044*	
O11W	1.1438 (4)	0.6187 (2)	0.7811 (2)	0.0317 (6)	
H21W	1.1856	0.5531	0.8238	0.048*	
H22W	1.0735	0.6526	0.8170	0.048*	
O9W	0.3769 (4)	0.3668 (2)	0.7147 (2)	0.0367 (7)	
H17W	0.5013	0.3639	0.6899	0.055*	
H18W	0.3103	0.3295	0.6970	0.055*	
O10W	0.2681 (5)	0.3942 (3)	0.9122 (2)	0.0438 (8)	
H19W	0.2749	0.3769	0.8592	0.066*	
H20W	0.3833	0.4069	0.9185	0.066*	
O7W	0.8872 (5)	0.7396 (3)	0.8714 (3)	0.0496 (8)	
H14W	0.9288	0.8041	0.8520	0.074*	
H13W	0.8565	0.7050	0.9407	0.074*	
O8W	0.7517 (5)	0.9492 (3)	0.5860 (2)	0.0424 (7)	
H15W	0.7651	0.9794	0.6271	0.064*	
H16W	0.7585	0.9966	0.5189	0.064*	

### Atomic displacement parameters (Å^2^)

**Table:**

	*U*^11^	*U*^22^	*U*^33^	*U*^12^	*U*^13^	*U*^23^
C1	0.029 (2)	0.0227 (18)	0.0295 (18)	−0.0097 (16)	−0.0020 (15)	−0.0105 (15)
C2	0.0208 (18)	0.0251 (18)	0.0231 (16)	−0.0018 (14)	−0.0042 (14)	−0.0103 (14)
C3	0.0242 (19)	0.0216 (17)	0.0278 (17)	−0.0024 (15)	−0.0073 (14)	−0.0072 (15)
C4	0.0229 (19)	0.0274 (19)	0.0193 (16)	−0.0061 (15)	−0.0060 (14)	−0.0035 (15)
C5	0.024 (2)	0.0290 (19)	0.0247 (17)	−0.0032 (15)	−0.0048 (14)	−0.0123 (15)
C6	0.0212 (18)	0.0256 (18)	0.0238 (16)	−0.0049 (15)	−0.0054 (14)	−0.0097 (15)
C7	0.0190 (18)	0.0250 (18)	0.0237 (17)	−0.0026 (14)	−0.0076 (14)	−0.0083 (14)
C8	0.027 (2)	0.0215 (17)	0.0224 (16)	−0.0049 (15)	−0.0060 (14)	−0.0086 (14)
C9	0.027 (2)	0.0268 (18)	0.0219 (16)	−0.0035 (16)	−0.0058 (15)	−0.0102 (15)
N1	0.0286 (18)	0.0352 (19)	0.0218 (15)	−0.0069 (15)	−0.0034 (13)	−0.0059 (14)
Na1	0.0375 (10)	0.0382 (9)	0.0305 (8)	−0.0049 (7)	−0.0042 (7)	−0.0110 (7)
Ni1	0.0249 (3)	0.0215 (3)	0.0229 (3)	−0.0038 (2)	−0.0044 (2)	−0.0076 (2)
O1	0.0469 (19)	0.0283 (14)	0.0402 (15)	0.0007 (13)	−0.0059 (13)	−0.0159 (13)
O2	0.0445 (18)	0.0354 (15)	0.0258 (13)	−0.0081 (13)	−0.0083 (12)	−0.0128 (12)
O3	0.0239 (14)	0.0325 (14)	0.0257 (12)	−0.0051 (11)	−0.0023 (10)	−0.0073 (11)
O4	0.0290 (15)	0.0288 (13)	0.0224 (12)	−0.0009 (11)	−0.0094 (10)	−0.0062 (11)
O5	0.0265 (14)	0.0211 (12)	0.0263 (12)	−0.0054 (10)	−0.0020 (10)	−0.0082 (10)
O6	0.0418 (18)	0.0303 (15)	0.0416 (16)	−0.0056 (13)	0.0098 (13)	−0.0165 (13)
O7	0.051 (2)	0.0521 (19)	0.0291 (14)	−0.0078 (16)	0.0046 (14)	−0.0131 (14)
O8	0.053 (2)	0.0287 (16)	0.0350 (15)	−0.0074 (14)	−0.0063 (14)	0.0010 (13)
O5W	0.054 (2)	0.068 (2)	0.0413 (17)	0.0225 (19)	−0.0073 (15)	−0.0142 (17)
O6W	0.0432 (19)	0.059 (2)	0.0490 (17)	−0.0078 (16)	−0.0051 (14)	−0.0310 (16)
O4W	0.0354 (16)	0.0297 (14)	0.0360 (14)	−0.0046 (12)	−0.0022 (12)	−0.0127 (12)
O2W	0.0330 (15)	0.0268 (13)	0.0237 (12)	−0.0077 (11)	−0.0051 (10)	−0.0068 (11)
O3W	0.0505 (19)	0.0242 (13)	0.0279 (13)	−0.0034 (12)	−0.0011 (12)	−0.0073 (11)
O1W	0.0297 (15)	0.0306 (14)	0.0255 (12)	−0.0022 (11)	−0.0057 (11)	−0.0093 (11)
O11W	0.0368 (16)	0.0323 (14)	0.0285 (13)	−0.0041 (12)	−0.0066 (11)	−0.0142 (11)
O9W	0.0270 (15)	0.0408 (16)	0.0450 (16)	0.0012 (13)	−0.0077 (12)	−0.0208 (14)
O10W	0.0432 (19)	0.0519 (18)	0.0344 (15)	−0.0023 (15)	−0.0077 (13)	−0.0164 (14)
O7W	0.074 (2)	0.0348 (16)	0.0421 (16)	−0.0121 (16)	0.0063 (16)	−0.0217 (14)
O8W	0.051 (2)	0.0383 (16)	0.0436 (16)	−0.0027 (14)	−0.0094 (14)	−0.0216 (14)

### Geometric parameters (Å, °)

**Table:**

C1—O1	1.251 (5)	Ni1—O1W	2.048 (3)
C1—O2	1.257 (4)	Ni1—O3W	2.053 (3)
C1—C2	1.520 (5)	Ni1—O2W	2.074 (3)
C2—C3	1.397 (5)	Ni1—O11W	2.100 (3)
C2—C7	1.402 (5)	Ni1—O4W	2.106 (3)
C3—C4	1.371 (5)	Ni1—Na1^ii^	3.4258 (18)
C3—H3	0.9300	O5W—H10W	0.8400
C4—C5	1.387 (5)	O5W—H9W	0.8400
C4—N1	1.471 (4)	O6W—H12W	0.8400
C5—C6	1.386 (5)	O6W—H11W	0.8400
C5—H5	0.9300	O4W—H8W	0.8400
C6—C7	1.407 (5)	O4W—H7W	0.8400
C6—C9	1.512 (5)	O2W—H3W	0.8400
C7—C8	1.523 (5)	O2W—H4W	0.8400
C8—O3	1.250 (4)	O3W—Na1^ii^	2.982 (3)
C8—O4	1.265 (4)	O3W—H5W	0.8400
C9—O6	1.261 (5)	O3W—H6W	0.8400
C9—O5	1.268 (4)	O1W—Na1^ii^	2.595 (3)
N1—O8	1.222 (4)	O1W—H1W	0.8400
N1—O7	1.225 (5)	O1W—H2W	0.8400
Na1—O5W	2.337 (4)	O11W—Na1^ii^	2.553 (3)
Na1—O6W	2.424 (4)	O11W—H21W	0.8400
Na1—O11W^i^	2.553 (3)	O11W—H22W	0.8400
Na1—O1W^i^	2.595 (3)	O9W—H17W	0.8401
Na1—O4W	2.611 (3)	O9W—H18W	0.8400
Na1—O2W	2.782 (3)	O10W—H19W	0.8400
Na1—O3W^i^	2.982 (3)	O10W—H20W	0.8401
Na1—Ni1^i^	3.4258 (18)	O7W—H14W	0.8400
Na1—H10W	2.6736	O7W—H13W	0.8400
Na1—H11W	2.5038	O8W—H15W	0.8400
Ni1—O5	2.032 (2)	O8W—H16W	0.8400
			
O1—C1—O2	126.1 (4)	O1W^i^—Na1—H11W	153.1
O1—C1—C2	116.1 (3)	O4W—Na1—H11W	78.9
O2—C1—C2	117.8 (3)	O2W—Na1—H11W	120.7
C3—C2—C7	119.5 (3)	O3W^i^—Na1—H11W	93.9
C3—C2—C1	118.8 (3)	Ni1^i^—Na1—H11W	120.3
C7—C2—C1	121.7 (3)	H10W—Na1—H11W	96.2
C4—C3—C2	119.4 (3)	O5—Ni1—O1W	89.70 (11)
C4—C3—H3	120.3	O5—Ni1—O3W	174.16 (10)
C2—C3—H3	120.3	O1W—Ni1—O3W	85.19 (11)
C3—C4—C5	122.4 (3)	O5—Ni1—O2W	85.04 (10)
C3—C4—N1	119.1 (3)	O1W—Ni1—O2W	97.31 (10)
C5—C4—N1	118.5 (3)	O3W—Ni1—O2W	92.77 (11)
C6—C5—C4	118.6 (3)	O5—Ni1—O11W	91.88 (10)
C6—C5—H5	120.7	O1W—Ni1—O11W	83.66 (10)
C4—C5—H5	120.7	O3W—Ni1—O11W	90.39 (11)
C5—C6—C7	120.3 (3)	O2W—Ni1—O11W	176.77 (10)
C5—C6—C9	118.7 (3)	O5—Ni1—O4W	93.85 (11)
C7—C6—C9	121.0 (3)	O1W—Ni1—O4W	175.04 (11)
C2—C7—C6	119.6 (3)	O3W—Ni1—O4W	91.42 (11)
C2—C7—C8	119.3 (3)	O2W—Ni1—O4W	86.46 (11)
C6—C7—C8	121.0 (3)	O11W—Ni1—O4W	92.76 (11)
O3—C8—O4	125.7 (3)	O5—Ni1—Na1^ii^	118.34 (8)
O3—C8—C7	118.2 (3)	O1W—Ni1—Na1^ii^	49.05 (8)
O4—C8—C7	116.1 (3)	O3W—Ni1—Na1^ii^	59.89 (9)
O6—C9—O5	125.0 (3)	O2W—Ni1—Na1^ii^	134.65 (8)
O6—C9—C6	117.9 (3)	O11W—Ni1—Na1^ii^	47.99 (8)
O5—C9—C6	117.1 (3)	O4W—Ni1—Na1^ii^	126.02 (9)
O8—N1—O7	123.8 (3)	C9—O5—Ni1	128.6 (2)
O8—N1—C4	118.2 (3)	Na1—O5W—H10W	104.5
O7—N1—C4	118.0 (3)	Na1—O5W—H9W	134.6
O5W—Na1—O6W	90.09 (13)	H10W—O5W—H9W	111.2
O5W—Na1—O11W^i^	146.60 (14)	Na1—O6W—H12W	144.4
O6W—Na1—O11W^i^	91.75 (11)	Na1—O6W—H11W	85.6
O5W—Na1—O1W^i^	90.25 (13)	H12W—O6W—H11W	111.8
O6W—Na1—O1W^i^	133.78 (12)	Ni1—O4W—Na1	104.27 (11)
O11W^i^—Na1—O1W^i^	65.02 (9)	Ni1—O4W—H8W	103.8
O5W—Na1—O4W	121.28 (13)	Na1—O4W—H8W	122.0
O6W—Na1—O4W	94.00 (11)	Ni1—O4W—H7W	96.9
O11W^i^—Na1—O4W	91.85 (10)	Na1—O4W—H7W	114.2
O1W^i^—Na1—O4W	124.33 (10)	H8W—O4W—H7W	111.5
O5W—Na1—O2W	75.95 (10)	Ni1—O2W—Na1	99.62 (11)
O6W—Na1—O2W	139.81 (12)	Ni1—O2W—H3W	101.7
O11W^i^—Na1—O2W	120.48 (10)	Na1—O2W—H3W	129.8
O1W^i^—Na1—O2W	84.54 (9)	Ni1—O2W—H4W	116.3
O4W—Na1—O2W	64.05 (9)	Na1—O2W—H4W	99.2
O5W—Na1—O3W^i^	84.67 (11)	H3W—O2W—H4W	110.8
O6W—Na1—O3W^i^	74.83 (10)	Ni1—O3W—Na1^ii^	83.57 (10)
O11W^i^—Na1—O3W^i^	63.77 (9)	Ni1—O3W—H5W	122.1
O1W^i^—Na1—O3W^i^	59.21 (9)	Na1^ii^—O3W—H5W	93.9
O4W—Na1—O3W^i^	152.24 (10)	Ni1—O3W—H6W	122.3
O2W—Na1—O3W^i^	138.79 (10)	Na1^ii^—O3W—H6W	114.5
O5W—Na1—Ni1^i^	109.49 (12)	H5W—O3W—H6W	111.2
O6W—Na1—Ni1^i^	101.28 (9)	Ni1—O1W—Na1^ii^	94.35 (11)
O11W^i^—Na1—Ni1^i^	37.68 (6)	Ni1—O1W—H1W	117.0
O1W^i^—Na1—Ni1^i^	36.60 (6)	Na1^ii^—O1W—H1W	104.3
O4W—Na1—Ni1^i^	126.75 (8)	Ni1—O1W—H2W	117.9
O2W—Na1—Ni1^i^	118.89 (8)	Na1^ii^—O1W—H2W	108.9
O3W^i^—Na1—Ni1^i^	36.54 (6)	H1W—O1W—H2W	111.6
O5W—Na1—H10W	17.7	Ni1—O11W—Na1^ii^	94.33 (10)
O6W—Na1—H10W	93.1	Ni1—O11W—H21W	110.8
O11W^i^—Na1—H10W	129.0	Na1^ii^—O11W—H21W	110.9
O1W^i^—Na1—H10W	75.6	Ni1—O11W—H22W	107.3
O4W—Na1—H10W	138.2	Na1^ii^—O11W—H22W	120.5
O2W—Na1—H10W	84.7	H21W—O11W—H22W	111.5
O3W^i^—Na1—H10W	68.8	H17W—O9W—H18W	111.5
Ni1^i^—Na1—H10W	91.8	H19W—O10W—H20W	111.2
O5W—Na1—H11W	87.5	H14W—O7W—H13W	111.9
O6W—Na1—H11W	19.5	H15W—O8W—H16W	111.9
O11W^i^—Na1—H11W	104.4		

Symmetry codes: (i) *x*−1, *y*, *z*; (ii) *x*+1, *y*, *z*.

### Hydrogen-bond geometry (Å, °)

**Table:**

*D*—H···*A*	*D*—H	H···*A*	*D*···*A*	*D*—H···*A*
O8W—H16W···O6W^iii^	0.84	2.07	2.876 (4)	161
O8W—H15W···O2^iv^	0.84	2.06	2.835 (4)	153
O7W—H13W···O10W^v^	0.84	1.93	2.748 (4)	163
O7W—H14W···O1^iv^	0.84	1.90	2.731 (4)	172
O10W—H20W···O6	0.84	1.92	2.735 (5)	165
O10W—H19W···O9W	0.84	2.01	2.813 (4)	160
O9W—H18W···O3^i^	0.84	1.99	2.802 (4)	161
O9W—H17W···O4	0.84	1.89	2.734 (4)	176
O11W—H22W···O7W	0.84	1.85	2.676 (4)	168
O11W—H21W···O10W^ii^	0.84	1.95	2.784 (4)	173
O1W—H2W···O4^vi^	0.84	1.89	2.721 (3)	172
O1W—H1W···O9W^ii^	0.84	1.86	2.685 (4)	168
O3W—H6W···O8W	0.84	1.85	2.660 (4)	162
O3W—H5W···O2^vi^	0.84	1.97	2.783 (4)	162
O2W—H4W···O3^vi^	0.84	1.83	2.657 (3)	170
O2W—H3W···O4	0.84	2.00	2.826 (4)	168
O4W—H7W···O6	0.84	1.84	2.655 (4)	163
O4W—H8W···O7W	0.84	2.02	2.801 (4)	154
O6W—H11W···O8W	0.84	2.09	2.900 (5)	161
O6W—H12W···O1^vii^	0.84	2.11	2.919 (4)	160
O5W—H9W···O3^vi^	0.84	2.25	2.935 (4)	139
O5W—H10W···O2^viii^	0.84	2.18	2.913 (5)	145

Symmetry codes: (iii) −*x*+1, −*y*+2, −*z*+1; (iv) *x*, *y*+1, *z*; (v) −*x*+1, −*y*+1, −*z*+2; (i) *x*−1, *y*, *z*; (ii) *x*+1, *y*, *z*; (vi) −*x*+2, −*y*+1, −*z*+1; (vii) *x*−1, *y*+1, *z*; (viii) −*x*+1, −*y*+1, −*z*+1.
